# Fatigue as a moderator in symptom networks of insomnia, anxiety, and depression: insights from moderated network analysis

**DOI:** 10.3389/fpsyt.2025.1644015

**Published:** 2025-12-29

**Authors:** Hanyue Xing, Xu Ma, Fanqiang Meng, Zhanjiang Li

**Affiliations:** Beijing An Ding Hospital Beijing Key Laboratory of Neuropsychopharmacology, Beijing, China

**Keywords:** fatigue, insomnia, anxiety, depression, moderate, network

## Abstract

**Background:**

Insomnia is closely associated with anxiety and depression, forming a complex bidirectional relationship. Although previous research has demonstrated that fatigue is a core and bridge symptom within this complex relationship, its potential moderating role in their interaction remains unexplored. This study employs symptom network analysis to explore the Moderating role of fatigue, aiming to identify core symptoms and their interactions.

**Methods:**

A total of 544 participants (mean age 37.86 ± 11.41 years, 210 males) diagnosed with chronic insomnia disorder were included. Sleep quality was assessed using the Pittsburgh Sleep Quality Index (PSQI), insomnia severity with the Insomnia Severity Index (ISI), anxiety with the Hamilton Anxiety Scale (HAMA), depression with the 17-item Hamilton Depression Rating Scale (HAMD-17), and fatigue with the 14-item Fatigue Scale (FS-14). Hierarchical LASSO was applied to estimate symptom networks, with total scores from the FS-14 used to measure fatigue as the moderating variable.

**Results:**

Our analysis revealed significant bidirectional relationships among symptoms of insomnia, depression, and anxiety. Fatigue played a key moderating role, particularly in the relationships linking difficulty staying asleep with cognitive impairment, somatic anxiety with daytime dysfunction, and difficulty staying asleep with circadian rhythm. Centrality analysis identified mental anxiety, the impact of insomnia on quality of life, and sleep-related interference with daytime function as core symptoms in the network.

**Conclusion:**

We identified significant bidirectional relationships between the symptoms of insomnia, depression, and anxiety symptoms and interaction terms moderated by fatigue. These findings provide valuable theoretical and practical insights for disrupting the cycle of these interconnected symptoms through targeted interventions addressing fatigue.

## Introduction

Insomnia disorder is the most common type of sleep disorder (1). The reported prevalence of insomnia among adults ranges widely, with estimates spanning from 10% to upwards of 40% (2–4). The prevalence of insomnia among the general population in China is approximately 15% (5). Insomnia not only causes significant distress to patients but also has a high comorbidity rate with anxiety and depression (6–8). Research shows that there is a complex bidirectional relationship between insomnia, anxiety, and depression (9–11). This interaction makes the diagnosis and treatment of these conditions more complicated.

The comorbidity of insomnia with anxiety and depression is extremely common (7). The clinical challenge lies in the intricate interplay of insomnia, anxiety, and depression (12). Current assessment tools are limited to measuring individual symptom severity but fail to quantify the strength of dynamic interactions between symptoms. Specifically, the limitations of previous studies primarily stem from two key aspects: first, inadequacies in research tools, and second, deficiencies in intervention studies. These shortcomings are fundamentally attributed to the failure to capture the complex interconnections between symptoms—research tools are constrained to evaluating isolated symptom severity rather than the intricate dynamic interactions among symptoms, and intervention studies thus lack a basis for identifying centrally located, impactful targets within the symptom network. Conventional therapeutic approaches typically address these conditions separately. Notably, standard Cognitive Behavioral Therapy for Insomnia (CBT-I) demonstrates reduced remission rates and higher residual symptom rates when treating insomnia comorbid with anxiety and depression compared to managing uncomplicated insomnia (13, 14). This phenomenon arises from symptom interactions that mutually reinforce through feedback loops. (9, 11). For instance, sleep disturbances can exacerbate negative emotions, while anxiety and depression can further disrupt sleep architecture. This circular relationship forms a “vicious cycle” that is difficult to break with conventional single-target interventions. Moreover, this comorbidity masks underlying pathophysiological mechanisms, making it challenging to identify effective therapeutic targets (15).

To address this challenge, the symptom network analysis method has emerged and is widely applied in research in the field of psychiatry. Symptom network analysis posits that the symptoms of psychopathology are causally related through numerous biological, psychological, and social mechanisms (15). If these causal relationships are strong enough, the symptoms can generate a certain degree of feedback and thus sustain themselves. This method constructs a complex system by regarding symptoms as nodes and representing the associations between symptoms as edges in the network. This framework provides a new perspective for explaining the phenomenon of disease comorbidity and helps identify core symptoms and their interactions (15–17).

Previous network studies have highlighted fatigue as a central bridge between insomnia, anxiety, and depression. For example, fatigue correlates with both sleep maintenance difficulties and somatic anxiety (11, 18, 19). However, fatigue is not just a passive component of this network; it also acts as a plausible moderator that regulates the strength of connections between other symptoms. This role is supported by three clinically and theoretically grounded mechanisms, all aligned with the core targets of Cognitive Behavioral Therapy for Insomnia (CBT-I) (20, 21). First, fatigue reduces the cognitive resources people need to manage sleep-related worries that CBT-I explicitly trains (13, 20). When fatigued, individuals may find it harder to use CBT-I techniques like thought challenging or emotion regulation. Second, fatigue often leads to unhelpful behaviors that CBT-I specifically aims to reduce—such as daytime napping, staying in bed when awake, or cutting back on physical activity (22, 23). For instance, a fatigued individual might take a 2-hour afternoon nap to cope with tiredness; this nap then lowers their nighttime sleep drive, making it harder to fall asleep at night and worsening insomnia. Fatigue also reduces motivation to complete CBT-I homework (e.g., keeping a sleep diary or practicing relaxation exercises), which weakens the therapy’s ability to correct these unhelpful habits. Finally, fatigue impairs daytime productivity and quality of life, which amplifies distress about insomnia (3, 24, 25). When people feel too fatigued to work, or enjoy daily activities, they may grow more anxious about their sleep problems or feel discouraged about their ability to recover. These emotions further disrupt sleep and feed into depression symptoms, creating a loop where fatigue, insomnia, and mood symptoms reinforce each other.

However, no research has systematically examined how fatigue modulates the network structure of insomnia–anxiety–depression comorbidity. While traditional symptom networks capture partial correlations between symptoms, they are limited when moderators are involved. To address this limitation, Moderated Network Analysis (MNM) has been developed. MNM is based on nodewise regression and enables researchers to examine how the association between two symptoms is influenced by a third variable, thereby capturing complex moderation effects within psychological networks (17, 26, 27). This methodological flexibility makes MNM particularly suitable for our research question, as it allows us to test whether fatigue systematically alters the symptom-level interactions underlying insomnia, anxiety, and depression comorbidity.

This study applies the MNM to insomnia comorbidity for the first time, systematically characterizing symptom network architecture across varying fatigue states. Fatigue was treated as a moderating variable, and MNM (28) was employed to explore the symptom network structure of insomnia, anxiety, and depression. The primary objectives were to identify central symptoms and fatigue-modulated pivotal connections, thereby informing prioritized intervention targets for improving Cognitive Behavioral Therapy for Insomnia (CBT-I).

## Methods

### Participants

Our study protocol and consent form were approved by the Human Research and Ethics Committee of Xuanwu Hospital, which is affiliated to the Capital Medical University. All participants signed an informed consent form and the study was submitted to the Chinese Clinical Trial Registry (Registration number: ChiCTR2200063665).

A cohort of 544 participants (210 males, 334 females) diagnosed with insomnia disorder was enrolled in this study. Recruitment occurred through the outpatient and inpatient services of Beijing Anding Hospital, Capital Medical University, from October 2022 to December 2024. The inclusion criteria required subjects to present with primary sleep-related complaints as their chief medical concern (per DSM-5 criteria:≥3months of sleep difficulty, daytime impairment, not attributable to substances or medical conditions). All participants were assessed via the Mini International Neuropsychiatric Interview (MINI) 7.0, a validated diagnostic tool for psychiatric disorders. Specifically, the MINI 7.0 assessment results indicated that among these participants, 34 individuals met the formal diagnostic criteria for major depressive disorder, 130 individuals met the criteria for anxiety disorder, and 90 individuals had comorbid major depressive disorder and anxiety disorder. Exclusion criteria eliminated individuals with major psychiatric disorders (e.g., schizophrenia, bipolar disorder) or serious somatic conditions. All participants provided written informed consent prior to study inclusion.

### Measures

#### The Pittsburgh sleep quality index

The scale consists of 9 questions, covering aspects such as subjective sleep quality, sleep latency, sleep duration, sleep efficiency, sleep disturbances, use of sleeping medications and daytime dysfunction. It is used to assess the sleep quality of the subjects in the most recent one-month period. Comprising 18 items, these items are grouped into 7 components. Each component is scored on a scale of 0 - 3. The total PSQI score is obtained by cumulative scoring, with a total score range from 0 to 21 points. The higher the score, the poorer the sleep quality (29).

#### Insomnia severity index

The ISI consists of 7 items, which assess the severity of sleep-onset and sleep maintenance difficulties (including night-time and early-morning awakenings), satisfaction with the current sleep pattern, interference with daily functioning, the significance of impairment attributed to sleep problems, and the degree of distress or worry caused by sleep problems. Each item is scored on a scale of 0-4, and the total score ranges from 0 to 28. The higher the score, the more severe the insomnia (30).

#### Epworth sleepiness scale

The Epworth Sleepiness Scale was developed by Murray Johns from the Epworth Hospital in Melbourne, Australia in 1991. It is a commonly used self-assessment tool for measuring the degree of sleepiness. The scale includes 8 daily-life scenarios, such as sitting and reading, watching TV, and riding in a vehicle. Subjects are required to rate, on a scale of 0-3, the likelihood of dozing off in these scenarios. A score of 0 indicates “never”, and a score of 3 indicates “very likely”. The scores for each scenario are summed up to obtain the total score, which ranges from 0 to 24. The higher the score, the more severe the sleepiness. Generally, a score of over 10 suggests abnormal sleepiness (31).

#### The morningness-eveningness questionnaire-5 item

MEQ-5 is a simplified version of the classic MEQ, which is used to assess an individual’s circadian rhythm preference, namely morning-type or evening-type. The MEQ-5 consists of only 5 items, focusing on key issues such as wake-up time, energy levels at different times of the day, and the best working time. Participants respond according to their actual situations. Different options correspond to different scores. The total score reflects the type of circadian rhythm. Scores of 4–7 indicate Definite Evening-type, 8–11 Moderate Evening-type, 12–17 Intermediate-type, 18–21 Moderate Morning-type, and 22–25 Definite Morning-type (32).

#### Fatigue scale-14

The Fatigue Scale-14 was developed by Chalder et al. It is a commonly used scale for assessing the level of fatigue. The scale contains 14 items, covering both physical and mental dimensions. In the physical aspect, it involves issues such as muscle weakness and decline in physical strength. Mentally, it includes difficulties in concentrating and memory impairment. Subjects make judgments on relevant questions based on their conditions in the past month, choosing either “about the same as before, or better” or “worse than before”. The scores of all items are added up to obtain the total score. The higher the score, the more severe the fatigue (33).

#### The Hamilton anxiety scale

HAMA is a classic clinician- rated scale for assessing the state of anxiety in clinical practice. The scale consists of 14 items, covering dimensions such as anxious mood, tension, fear, and insomnia, providing a comprehensive representation of anxiety symptoms. It adopts a 5-point rating scale from 0 to 4. Psychiatrists score based on the patient’s performance in the past week. “0” represents no symptoms, and “4” represents extremely severe symptoms. The higher the score, the more severe the degree of anxiety (34).

#### The Hamilton depression rating scale

HAMD-17 is a classic scale for evaluating the state of depression. The scale encompasses 17 items, covering multiple dimensions such as affective disorders, cognitive impairments, sleep disorders, and somatic symptoms. Common depressive manifestations like depressive mood, self-reproach and guilt, difficulty falling asleep, and loss of appetite are all within the scope of assessment. A 5-point rating scale from 0 to 4 is adopted. Psychiatrists score according to the patient’s actual performance in the past week. “0” indicates no symptoms, while “4” indicates extremely severe symptoms. The higher the score, the more severe the degree of depression(M) (35).

### Statistical analysis

R was used to perform all statistical analyses (version 4.4.2). Participants with more than 20% missing data on assessment scales were excluded based on predefined criteria, resulting in the removal of 82 cases. Multiple imputation was then performed on the remaining sample using the mice package (36) to handle the remaining missing values. The default imputation methods in mice were applied, including predictive mean matching for continuous variables. This approach has been adopted in previous network analysis studies (37–40).

### Network estimation

To overcome the limitations of traditional variable selection methods (e.g., LASSO) in capturing higher-order interactions within moderated network models (MNMs), we adopted the hierarchical LASSO approach (41). This method retains all relevant lower-order terms when higher-order interactions are included. Variable selection was performed using the *varSelect* function in the *modnets* package, and network estimation was conducted via the *fitNetwork* function.

For network visualization, we applied the OR rule for edge inclusion. Specifically, an edge between two nodes was retained if at least one of the corresponding nodewise regressions showed a significant main or interaction effect at *p* <.05. Solid edges indicate significant main (non-interaction) effects between two nodes, while dashed edges indicate that the relationship includes a significant moderation by O3. Edge thickness corresponds to the magnitude of the standardized regression coefficients, with green lines representing positive associations and red lines representing negative associations. Node importance was quantified using the expected influence index (42), which captures the sum of both positive and negative edge weights connected to each node.

### Network stability

To evaluate the stability of node centrality indices, we computed correlation stability coefficients (CS-coefficients) using case-dropping subset bootstraps. The bootNet function from the modnets package was used to estimate all stability metrics. A CS-coefficient greater than 0.25 is considered acceptable, while values above 0.5 indicate better stability (26).

## Results

### Descriptive statistics of sample characteristics

**Table 1** presents descriptive statistics for the components of insomnia, depression, anxiety, and other related symptoms, as well as sociodemographic information for the entire sample.

**Table 1:** Descriptive statistics for the components of insomnia, depression, anxiety, and other related symptoms for full sample.

Variables	Mean	SD	Median	Skewness	Kurtosis
age	37.86	11.41	37	0.36	–0.57
S1	1.88	1.22	2	0.01	–0.99
S2	1.45	1.14	1	0.4	–0.74
S3	1.6	1.22	2	0.17	–1.12
S4	2.59	1.06	3	–0.46	–0.55
S5	2.21	1.15	2	–0.15	–0.88
S6	2.28	1.12	2	–0.21	–0.78
S7	2.28	1.19	2	–0.10	–0.98
S8	1.88	0.79	2	–0.15	–0.65
O1	2.47	0.98	3	–1.58	0.94
O2	5.77	4.44	5	0.78	0.47
O3	6.02	5	5	0.36	–1.29
O4	14.33	4.19	14	0.03	–0.68
D1	1.15	1.41	1	1.25	0.96
D2	0.41	0.75	0	1.92	3.89
D3	0.07	0.31	0	5.01	25.33
D4	0.3	0.54	0	1.74	2.43
A1	3.6	2.76	4	0.58	0.22
A2	1.9	2.67	1	1.92	4.98

S1–S8: insomnia-related items; O1–O4: daytime and circadian-related symptoms; D1–D4: depressive symptoms; A1–A2: anxiety symptoms. Specifically: S1 = difficulty falling asleep; S2 = difficulty staying asleep; S3 = problem waking up too early in the morning; S4 = satisfaction with current sleep pattern; S5 = interference with daily functioning; S6 = significance of impairment attributed to sleep problems; S7 = degree of distress or worry caused by sleep problems; S8 = sleep quality; O1 = daytime dysfunction; O2 = sleepiness; O3 = fatigue; O4 = circadian rhythm; D1 = retardation; D2 = cognitive impairment; D3 = loss of weight; D4 = hypochondria; A1 = mental anxiety; A2 = somatic anxiety.

### Description of the network

The definitions of all nodes included in the network analysis are summarized in **Table 2**. To provide a clearer understanding of how fatigue (O3) influences the network, we first describe the overall network structure, followed by the moderation effects of O3 and the examination of centrality and stability indices.

**Table 2:** Definition of nodes included in the network analysis.

Node	Symptom description	Scale	Item(s)
S1	Difficulty falling asleep	ISI	Item 1A
S2	Difficulty staying asleep	ISI	Item 1B
S3	Waking up too early	ISI	Item 1C
S4	Satisfaction with current sleep pattern	ISI	Item 2
S5	Interference with daily functioning	ISI	Item 3
S6	Significance of impairment due to sleep problems	ISI	Item 4
S7	Distress or worry caused by sleep problems	ISI	Item 5
S8	Sleep quality	PSQI	Dimension 1
A1	Mental anxiety	HAMA	Items 1, 2, 3, 5, 14
A2	Somatic anxiety	HAMA	Items 7–13
D1	Retardation	HAMD-17	Items 1, 7, 8, 14
D2	Cognitive impairment	HAMD-17	Items 2–3
D3	Weight loss	HAMD-17	Item 16
D4	Hypochondriasis	HAMD-17	Item 15
O1	Daytime dysfunction	PSQI	Dimension 7
O2	Sleepiness	Epworth Sleepiness Scale	Total score
O3	Fatigue	Chalder Fatigue Scale-14	Total score
O4	Circadian rhythm preference	MEQ-5	Total score

**Figure 1:**
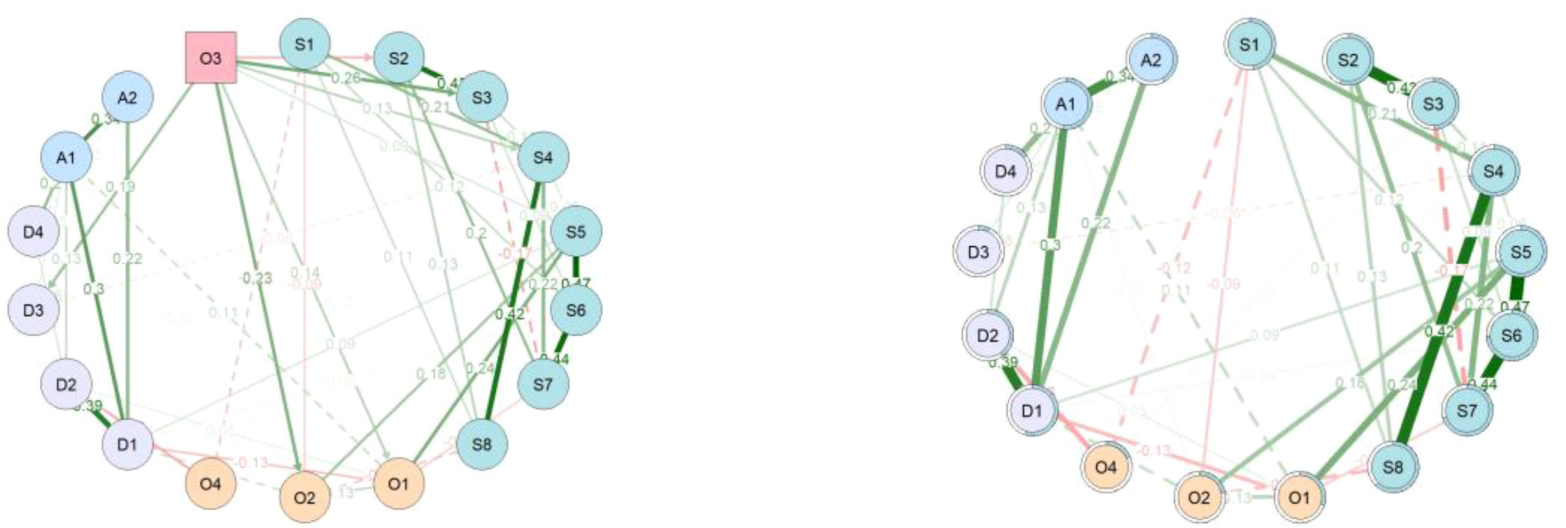
Visualization of the moderated network model. The left panel shows the network including the exogenous moderator O3 (red square), and the right panel shows the same network excluding O3 for clarity. The light blue shading of each node represents the R² value from its nodewise regression. Solid lines indicate significant main (non-interaction) associations between nodes (p <.05), while dashed lines indicate significant moderation effects by O3 (p <.05), based on the OR rule applied across nodewise regressions. Green edges denote positive associations, and red edges denote negative associations. Edge thickness is proportional to the absolute value of the standardized regression coefficients.

This study conducted a preliminary screening of research variables by integrating existing literature with the clinical practical experience of two senior psychiatrists, and subsequently utilized the Varselect function from the Modnets package to finalize the selected variables (**Supplementary Table S1**, **Supplementary Figure S1**). As shown in **Figure 1**, the network model constructed using hierarchical LASSO revealed significant bidirectional relationships between insomnia, depression, anxiety, and other related symptoms. The nodewise regression matrix (**Supplementary Table S2**) demonstrated bidirectional and mutually predictive associations among insomnia symptoms, depressive symptoms, anxiety symptoms, and other related symptoms (9, 18, 19, 43). Significant associations also exist between different symptom dimensions, such as S5 (interference with daily functioning) with O2 (Sleepiness), and O4 (circadian rhythm) with D2 (Cognitive impairment).

### Moderation effects of fatigue

**Figure 2:**
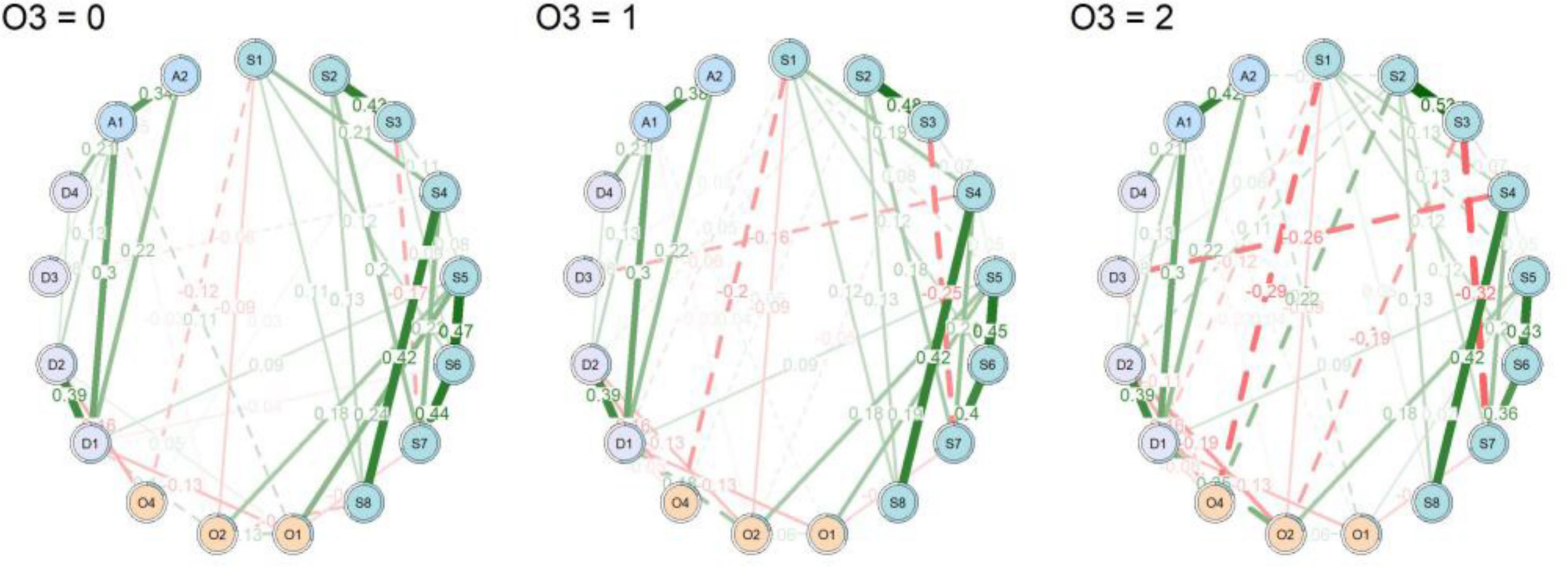
Conditional network models at the mean, +1 SD, and +2 SD levels of O3. The thickness and color of the edges represent the strength and direction of the conditional associations between nodes (green = positive, red = negative). The dashed line indicates that at least one of the interactive relationships between two nodes is moderated by the O3 variable at p <.05 (i.e., OR rule). As O3 levels increase, the network becomes more densely connected. Node labels correspond to symptom indicators shown in **Table 2**.

As shown in **Figure 2** and **Supplementary Table S3**, the moderation analysis revealed that O3 (fatigue) significantly moderated associations between insomnia, depression, anxiety, and other related symptoms. In insomnia and related symptoms, elevated O3 levels strengthened the positive predictive relationship between S2 (Difficulty staying asleep) and O4 (circadian rhythm).

**Figure 3:**
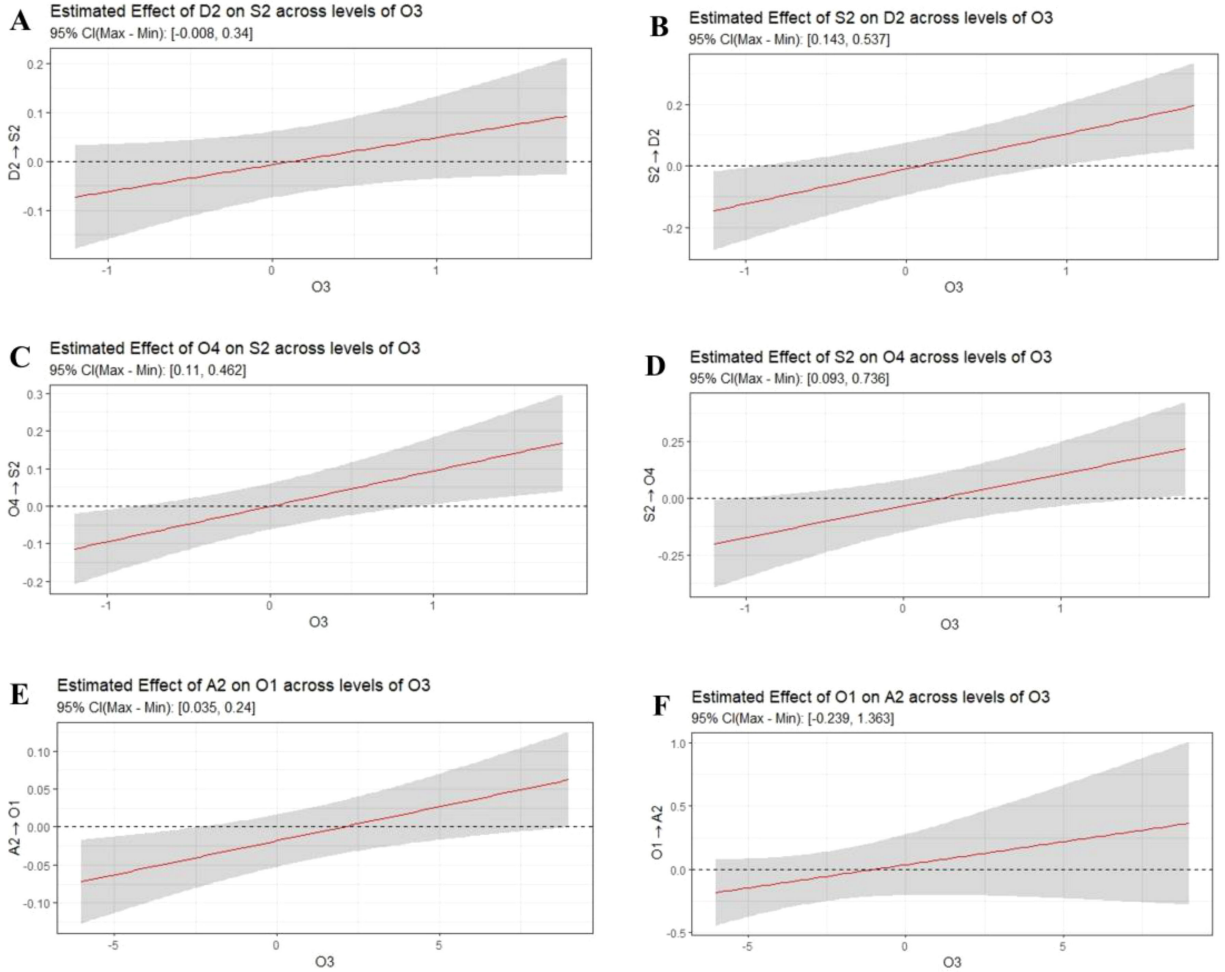
The plots of conditional marginal effects. Each plot represents average values across both relevant interaction terms. In **(A)**, the plot shows the conditional marginal effects of D2 × O3 on S2. In **(B)**, the plot shows the conditional marginal effects of S2 × O3 on D2. In **(C)**, the plot shows the conditional marginal effects of O4 × O3 on S2. In **(D)**, the plot shows the conditional marginal effects of S2 × O3 on O4. In **(E)**, the plot shows the conditional marginal effects of A2 × O3 on O1. In **(F)**, the plot shows the conditional marginal effects of O1 × O3 on A2.

Within emotion-related symptoms, O3 negatively moderated the association between S2 (difficulty staying asleep) and D2 (loss of weight), as illustrated in **Figures 3A, B**. Moreover, O3 significantly amplified the effect of A2 (somatic anxiety) on O1 (daytime dysfunction), as illustrated in **Figures 3E, F**.

We can now more clearly see how the network structure changes at increasing levels of O3 (see **Figure 3**). Looking only at the plots where thresholding was applied, we can see that some relations, between D3 (loss of weight) and S4 (satisfaction with the current sleep pattern), S1 (difficulty falling asleep) and O4 (circadian rhythm), and S3 (problem waking up too early in the morning) and S7 (the degree of distress or worry caused by sleep problems), remain relatively constant across levels of O3 among insomnia, depression, anxiety, and other related symptoms, spanning [-0.06, -0.26; -0.12, -0.29; -0.17, -0.32]. While some relations, between S2 (difficulty staying asleep) and O4 (circadian rhythm), and S2 (difficulty staying asleep) and D2 (cognitive impairment), exhibit a significant positive strengthening as O3 increases, as reflected by the significant interaction effects that modulate the strength of the edge, spanning [0, 0.22; 0, 0.11]. On the other hand, some relations, between S1 (difficulty falling asleep) and D1 (retardation) and S3 (problem waking up too early in the morning) and O2 (sleepiness), exhibit a significant negative strengthening as O3 increases. This is reflected by the significant interaction effects that modulate the strength of the edge, with values spanning [-0.06, -0.12] and [-0.05, -0.19], respectively.

### Network centrality, stability, and replication

In terms of centrality metrics, A1 (mental anxiety), S6 (the significance of impairment attributed to sleep problems), and S5 (interference with daily functioning) exhibited the highest Expected Influence (EI) values in the network model (**Supplementary Figure S2**), indicating their core roles in the network and stronger influences on other symptoms.

The stability of network parameters was evaluated using case-dropping subset bootstrap analysis (**Supplementary Figure S3**). As **Table 3** shown, the CS value for pairwise edge weights is.44, indicating that even when we dropped 75% of the total sample, at least 95% of the subsamples had pairwise edge weights that were correlated with those in the original sample at a value greater than 0.70. For interaction estimates, these values are 0.28. This suggests that the maximum drop size where a correlation of 0.70 was maintained across 95% of the subsamples was 28%. Overall, the present dataset performs well on most metrics, with coefficients for EI both above 0.25 for all pairwise weights and interaction estimates, suggesting that the network structure and symptom relationships remain relatively stable even under sample variability.

**Table 3:** Correlation stability coefficients.

Correlation stability CS (P_.95_) ≥.70		
Type	Edge	EI
Pairwise	0.44	0.52
Interactions	0.28	0.28

EI, Expected Influence.

## Discussion

This study investigates the bidirectional relationship between insomnia, anxiety, depression, and other related symptoms, taking into account fatigue in patients with insomnia disorder. To our knowledge, it is the first study to use the Moderated Network Model (MNM) to analyze insomnia, anxiety, depression, and other related symptoms in a sample of 544 patients with insomnia disorder. Our analysis revealed several important findings that need to be discussed in detail.

### The bidirectional relationship between insomnia, anxiety, depression, and other related symptoms

There are significant bidirectional relationships among insomnia, depression, anxiety, and other related symptoms. First, there is a strong association between anxiety (A1) and difficulty staying asleep (S2). For example, when patients lie awake ruminating about not sleeping, which further delays falling back asleep after nighttime awakenings.

Second, interference with daily functioning (S5) is closely associated with the perceived severity of impairment from sleep problems (S6). This suggests that greater disruption to daytime activities is linked to stronger perceptions of how much sleep issues harm quality of life. This is consistent with the conclusions of previous studies on the multi-dimensional impairment of the quality of life caused by sleep problems, such as the influence on work efficiency and the participation in social activities (44). Furthermore, S6 (the significance of impairment attributed to sleep problems) is closely linked to S7 (the degree of distress or worry caused by sleep problems), reflecting that the adverse impact of insomnia on the quality of life will increase the individual’s psychological burden regarding sleep (45, 46), forming a vicious cycle.

Additionally, there is a significant positive correlation between S5 (interference with daily functioning) and O2 (Sleepiness) (47). From a CBT-I perspective, this association highlights a key behavioral challenge: daytime sleepiness may lead to unhelpful coping behaviors (e.g., long afternoon naps, staying in bed when awake) that further disrupt nighttime sleep. For example, a patient who naps for 2 hours midday to manage sleepiness may then struggle to fall asleep at night, worsening insomnia and creating a loop of functional impairment, sleepiness, and poor sleep. This finding is of great significance for clinical practice, suggesting that doctors should pay attention to both the impairment of daytime function and the degree of sleepiness when evaluating insomnia patients, and adopt comprehensive intervention strategies, such as improving nighttime sleep quality, adjusting daytime activities, and helping patients establish healthy sleep habits through behavioral therapy.

Finally, our model finds a positive association between cognitive impairment (D2) and morningness chronotype (O4). This observation aligns with prior suggestions that patients with more depressive or negative cognition may be more likely to have a morning chronotype (48, 49). Especially for morningness individuals, it may be necessary to relieve their negative cognition and depressive mood through psychological support, stress management, or rhythm adjustment strategies, so as to improve their mental health status (45).

### The moderating effect of fatigue

Our analysis reveals that fatigue (previously labeled O3) is associated with changes in the strength of relationships between several key symptoms—findings with direct implications for CBT-I’s focus on personalized, symptom-targeted intervention.

Our model shows a positive association between difficulty staying asleep (S2) and morningness chronotype (O4), and this association is stronger in patients with higher fatigue. Specifically, among patients with high fatigue, morning-type individuals tend to report more severe difficulty staying asleep, while night-type individuals (who prefer later bedtimes/wake times) show smaller changes in this symptom.

Our model also finds that the positive association between difficulty staying asleep (S2) and cognitive impairment (D2) is stronger in patients with higher fatigue. This means that the link between struggling to stay asleep and experiencing negative thinking or poor concentration is more pronounced when fatigue is high. When fatigue is low, patients may have enough cognitive resources to cope with occasional sleep maintenance issues (e.g., using relaxation to fall back asleep, avoiding rumination about poor sleep). But as fatigue increases, these resources may become depleted: frequent nighttime awakenings could add to cognitive load, making negative thoughts more likely, while reduced emotional regulation capacity may amplify distress about sleep.

### The significance of network analysis in improving the CBT-I

Our findings suggest practical ways to improve CBT-I for patients with comorbid insomnia, anxiety, and depression by integrating fatigue management, focusing on core symptoms, and accounting for circadian rhythm differences. CBT for fatigue targets three interrelated dimensions—cognitive, behavioral, and emotional factors—that contribute to the maintenance of fatigue. Its core principle is to address maladaptive thoughts, behaviors, and emotional responses related to fatigue, rather than attempting to “eliminate” fatigue directly. At the cognitive level, patients are guided to identify and challenge catastrophic beliefs such as “fatigue equals physical fragility” or “activity worsens the condition.” These are replaced with evidence-based explanations—for example, understanding that short-term fatigue may reflect deconditioning that can improve through gradual activity. At the behavioral level, therapy involves developing progressive activity plans—similar to the “graded activity”. approach used in GET (Grade Exercise Therapy), but with greater emphasis on cognitive monitoring. Start with 10 minutes of light physical activity 3 times a week, increasing by 5 minutes weekly until reaching 30 minutes/session. Guide patients to map daily activities on a “fatigue-impact scale” (1 = low impact, 5 = high impact). Teach them to prioritize high-value, low-impact tasks (e.g., completing work reports in the morning when energy is higher) and schedule 5–10 minutes rest breaks after high-impact activities (e.g., 30 minutes of cleaning). This helps reduce cycles of excessive rest and sudden overexertion, thereby preventing fatigue accumulation. At the emotional level, CBT aims to reduce the amplifying effects of anxiety and depression on fatigue through relaxation training and emotional regulation techniques (50–52).

### Centrality, stability, and accuracy

Analyzed from the indicators of network centrality, stability and accuracy, A1 (mental anxiety), S6 (significance of impairment attributed to sleep problems), and S5 (interference with daily functioning) are in the core positions in the network model and have a greater impact on other symptoms. This indicates that in the comorbid symptom network, symptoms such as mental anxiety, the impact of insomnia on quality of life, and sleep interfering with daytime function play a key driving role in the entire symptom system, and may be important targets for breaking the vicious cycle of comorbidity.

The stability assessment shows that the data has good stability. This means that even if the sample changes, the network structure and the relationships between symptoms can still remain relatively stable, providing strong support for the reliability of the conclusions.

## Limitation

This study has certain limitations. There may be limitations in the sample selection. Samples were exclusively obtained from patients enrolled at Beijing Anding Hospital, a tertiary psychiatric hospital affiliated with Capital Medical University. It has not fully covered different regions, cultural backgrounds, and special populations, which may affect the broad representativeness of the results. Since this study adopts a cross-sectional design, it is difficult to clarify the causal and temporal relationships between symptoms, and it is impossible to accurately determine the sequence of the occurrence of symptoms and the dynamic process of their mutual influence.

In future research, the sample scope can be expanded to include people with different characteristics. A longitudinal research design can be adopted to track the changes in symptoms, and explore the causal relationships between symptoms in depth, so as to provide a more precise basis for clinical interventions.

## Conclusion

Despite these limitations, the data from this study still provides important clues for the research on the comorbidity of insomnia, anxiety, and depression. It has clarified the complex structure of the symptom network, the relationships between key symptoms, and the regulatory role of O3 (fatigue), laying a foundation for understanding the mechanism of comorbidity.

Based on these findings, subsequent research can further explore the mechanism of comorbidity, and seek precise intervention strategies targeting the core symptoms and key regulatory factors, so as to improve the current diagnosis and treatment situation for patients with insomnia comorbidity.

## Data Availability

The original contributions presented in the study are included in the article/**Supplementary Material**. Further inquiries can be directed to the corresponding author.
