# Supplementary figures and images for: Fatigue as a moderator in symptom networks of insomnia, anxiety, and depression: insights from moderated network analysis
Source: Front Psychiatry. 2025 Dec 29;16:1644015. doi: 10.3389/fpsyt.2025.1644015 (PMC12794031; doi:10.3389/fpsyt.2025.1644015)

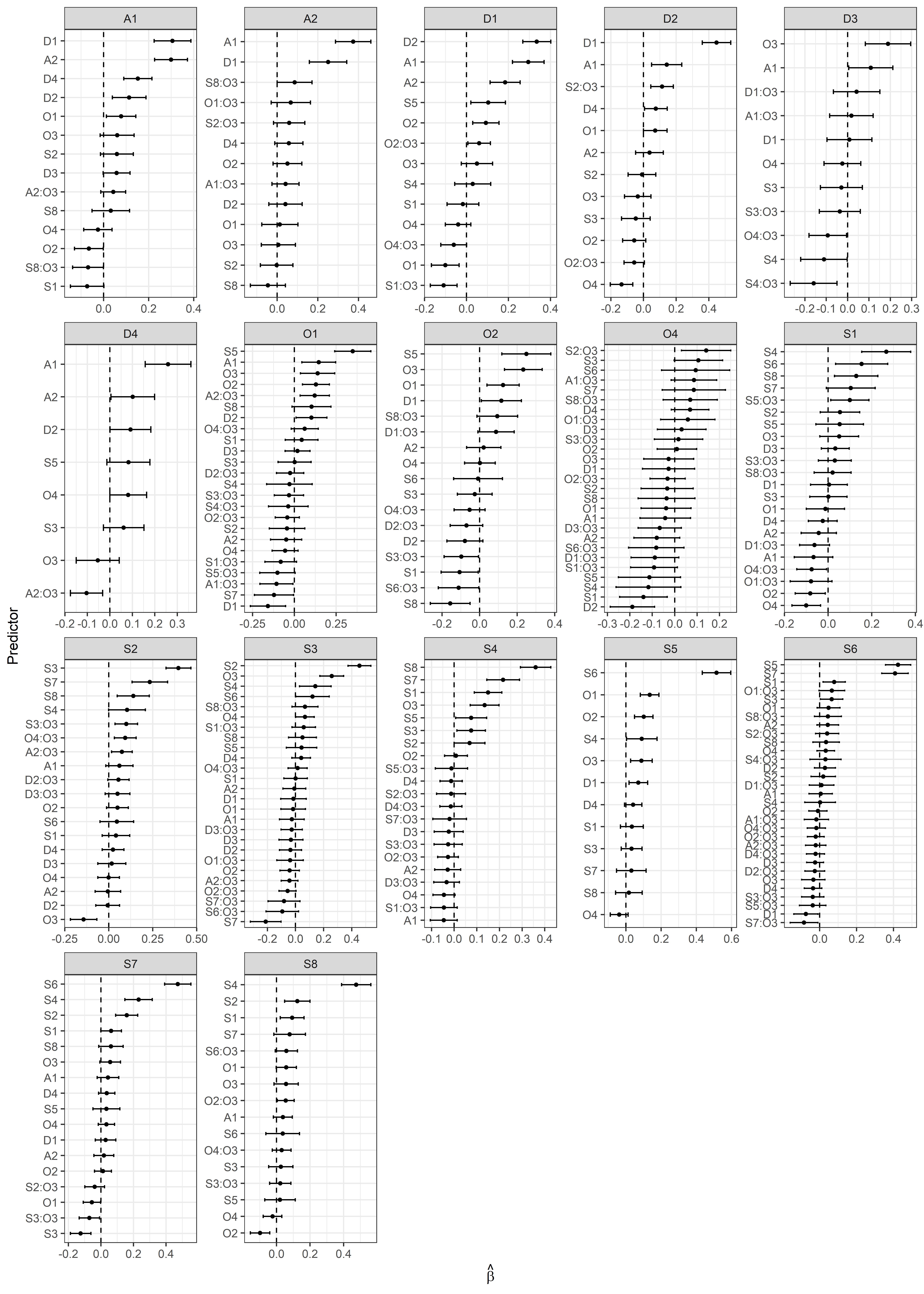

Supplement: Supplementary file 1 [file Image1.jpg]

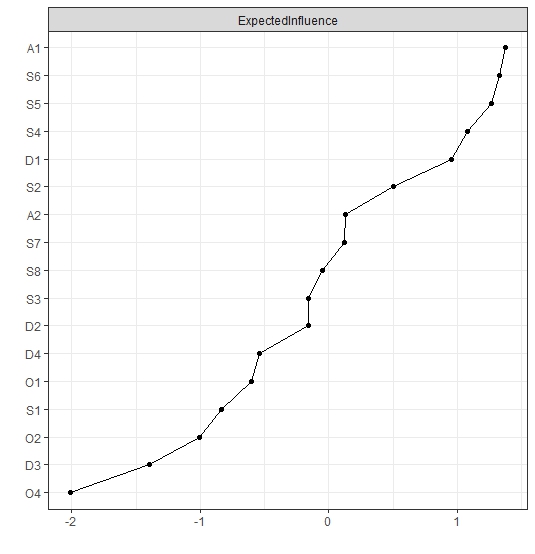

Supplement: Supplementary file 2 [file Image2.jpeg]

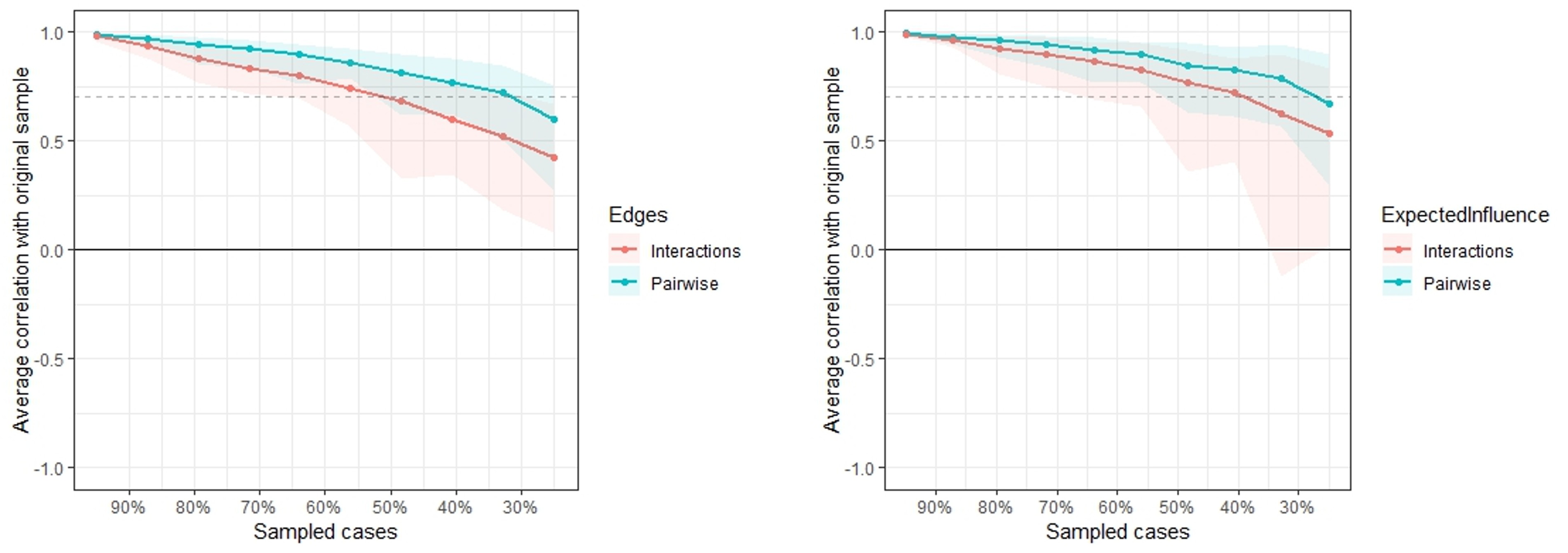

Supplement: Supplementary file 3 [file Image3.jpg]
