# Supplementary material for: Fatigue as a moderator in symptom networks of insomnia, anxiety, and depression: insights from moderated network analysis
Source: Front Psychiatry. 2025 Dec 29;16:1644015. doi: 10.3389/fpsyt.2025.1644015 (PMC12794031; doi:10.3389/fpsyt.2025.1644015)
Supplement: Supplementary file 4 [file Table1.docx]

**Supplementary Table S1. Variables were selected through the varSelect function selection procedure.**

|  | S1 | S2 | S3 | S4 | S5 | S6 | S7 | S8 | O1 | O2 | O4 | D1 | D2 | D3 | D4 | A1 | A2 |
| --- | --- | --- | --- | --- | --- | --- | --- | --- | --- | --- | --- | --- | --- | --- | --- | --- | --- |
| mods | S2 | S1 | S1 | S1 | S1 | S1 | S1 | S1 | S1 | S1 | S1 | S1 | S2 | S3 | S3 | S1 | S2 |
|  | S3 | S3 | S2 | S2 | S3 | S2 | S2 | S2 | S2 | S3 | S2 | S4 | S3 | S4 | S5 | S2 | S8 |
|  | S4 | S4 | S4 | S3 | S4 | S3 | S3 | S3 | S3 | S5 | S3 | S5 | O1 | O4 | O4 | S8 | O1 |
|  | S5 | S6 | S5 | S5 | S6 | S4 | S4 | S4 | S4 | S6 | S4 | O1 | O2 | D1 | D2 | O1 | O2 |
|  | S6 | S7 | S6 | S7 | S7 | S5 | S5 | S5 | S5 | S8 | S5 | O2 | O4 | A1 | A1 | O2 | D1 |
|  | S7 | S8 | S7 | S8 | S8 | S7 | S6 | S6 | S7 | O1 | S6 | O4 | D1 | O3 | A2 | O4 | D2 |
|  | S8 | O2 | S8 | O2 | O1 | S8 | S8 | S7 | S8 | O4 | S7 | D2 | D4 |  | O3 | D1 | D4 |
|  | O1 | O4 | O1 | O4 | O2 | O1 | O1 | O1 | O2 | D1 | S8 | A1 | A1 |  |  | D2 | A1 |
|  | O2 | D2 | O2 | D3 | O4 | O2 | O2 | O2 | O4 | D2 | O1 | A2 | A2 |  |  | D3 | O3 |
|  | O4 | D3 | O4 | D4 | D1 | O4 | O4 | O4 | D1 | A2 | O2 | O3 | O3 |  |  | D4 |  |
|  | D1 | D4 | D1 | A1 | D4 | D1 | D1 | A1 | D2 | O3 | D1 |  |  |  |  | A2 |  |
|  | D3 | A1 | D2 | A2 | O3 | D2 | D4 | O3 | D3 |  | D2 |  |  |  |  | O3 |  |
|  | D4 | A2 | D3 | O3 |  | D3 | A1 |  | A1 |  | D3 |  |  |  |  |  |  |
|  | A1 | O3 | D4 |  |  | D4 | A2 |  | A2 |  | D4 |  |  |  |  |  |  |
|  | A2 |  | A1 |  |  | A1 | O3 |  | O3 |  | A1 |  |  |  |  |  |  |
|  | O3 |  | A2 |  |  | A2 |  |  |  |  | A2 |  |  |  |  |  |  |
|  |  |  | O3 |  |  | O3 |  |  |  |  | O3 |  |  |  |  |  |  |
| ints | S3:O3 | S3:O3 | S1:O3 | S1:O3 |  | S2:O3 | S2:O3 | S3:O3 | S1:O3 | S3:O3 | S1:O3 | S1:O3 | S2:O3 | S3:O3 | A2:O3 | S8:O3 | S2:O3 |
|  | S5:O3 | O4:O3 | S6:O3 | S2:O3 |  | S3:O3 | S3:O3 | S6:O3 | S3:O3 | S6:O3 | S2:O3 | O2:O3 | O2:O3 | S4:O3 |  | A2:O3 | S8:O3 |
|  | S8:O3 | D2:O3 | S7:O3 | S3:O3 |  | S4:O3 |  | O2:O3 | S4:O3 | S8:O3 | S3:O3 | O4:O3 |  | O4:O3 |  |  | O1:O3 |
|  | O1:O3 | D3:O3 | S8:O3 | S5:O3 |  | S5:O3 |  | O4:O3 | S5:O3 | O4:O3 | S6:O3 |  |  | D1:O3 |  |  | A1:O3 |
|  | O4:O3 | A2:O3 | O1:O3 | S7:O3 |  | S7:O3 |  |  | O2:O3 | D1:O3 | S8:O3 |  |  | A1:O3 |  |  |  |
|  | D1:O3 |  | O2:O3 | O2:O3 |  | S8:O3 |  |  | O4:O3 | D2:O3 | O1:O3 |  |  |  |  |  |  |
|  |  |  | O4:O3 | D3:O3 |  | O1:O3 |  |  | D2:O3 |  | O2:O3 |  |  |  |  |  |  |
|  |  |  | D3:O3 | D4:O3 |  | O2:O3 |  |  | A1:O3 |  | D1:O3 |  |  |  |  |  |  |
|  |  |  | A2:O3 |  |  | O4:O3 |  |  | A2:O3 |  | D3:O3 |  |  |  |  |  |  |
|  |  |  |  |  |  | D1:O3 |  |  |  |  | A1:O3 |  |  |  |  |  |  |
|  |  |  |  |  |  | D2:O3 |  |  |  |  |  |  |  |  |  |  |  |
|  |  |  |  |  |  | D4:O3 |  |  |  |  |  |  |  |  |  |  |  |
|  |  |  |  |  |  | A1:O3 |  |  |  |  |  |  |  |  |  |  |  |
|  |  |  |  |  |  | A2:O3 |  |  |  |  |  |  |  |  |  |  |  |
