# Supplementary material for: Fatigue as a moderator in symptom networks of insomnia, anxiety, and depression: insights from moderated network analysis
Source: Front Psychiatry. 2025 Dec 29;16:1644015. doi: 10.3389/fpsyt.2025.1644015 (PMC12794031; doi:10.3389/fpsyt.2025.1644015)
Supplement: Supplementary file 5 [file Table2.docx]

**Supplementary Table S2. The nodewise regression matrix for the moderated network model (*p* < .05).**

|  | S1 | S2 | S3 | S4 | S5 | S6 | S7 | S8 | O1 | O2 | O4 | D1 | D2 | D3 | D4 | A1 | A2 |
| --- | --- | --- | --- | --- | --- | --- | --- | --- | --- | --- | --- | --- | --- | --- | --- | --- | --- |
| S1 | 0.00 |  |  |  |  |  |  |  |  |  |  |  |  |  |  |  |  |
| S2 | 0.00 | 0.00 |  |  |  |  |  |  |  |  |  |  |  |  |  |  |  |
| S3 | 0.00 | 0.43 | 0.00 |  |  |  |  |  |  |  |  |  |  |  |  |  |  |
| S4 | 0.21 | 0.00 | 0.11 | 0.00 |  |  |  |  |  |  |  |  |  |  |  |  |  |
| S5 | 0.00 | 0.00 | 0.00 | 0.08 | 0.00 |  |  |  |  |  |  |  |  |  |  |  |  |
| S6 | 0.12 | 0.00 | 0.09 | 0.00 | 0.47 | 0.00 |  |  |  |  |  |  |  |  |  |  |  |
| S7 | 0.00 | 0.20 | -0.17 | 0.22 | 0.00 | 0.44 | 0.00 |  |  |  |  |  |  |  |  |  |  |
| S8 | 0.11 | 0.13 | 0.00 | 0.42 | 0.00 | 0.00 | 0.00 | 0.00 |  |  |  |  |  |  |  |  |  |
| O1 | 0.00 | 0.00 | 0.00 | 0.00 | 0.24 | 0.00 | -0.09 | 0.00 | 0.00 |  |  |  |  |  |  |  |  |
| O2 | -0.09 | 0.00 | 0.00 | 0.00 | 0.18 | 0.00 | 0.00 | -0.13 | 0.13 | 0.00 |  |  |  |  |  |  |  |
| O4 | -0.12 | 0.00 | 0.03 | 0.00 | 0.00 | 0.00 | 0.00 | 0.00 | 0.00 | 0.00 | 0.00 |  |  |  |  |  |  |
| D1 | 0.00 | 0.00 | 0.00 | 0.00 | 0.09 | -0.04 | 0.00 | 0.00 | -0.13 | 0.10 | 0.00 | 0.00 |  |  |  |  |  |
| D2 | 0.00 | 0.00 | 0.00 | 0.00 | 0.00 | 0.00 | 0.00 | 0.00 | 0.05 | 0.00 | -0.16 | 0.39 | 0.00 |  |  |  |  |
| D3 | 0.00 | 0.00 | 0.00 | -0.06 | 0.00 | 0.00 | 0.00 | 0.00 | 0.00 | 0.00 | 0.00 | 0.00 | 0.00 | 0.00 |  |  |  |
| D4 | 0.00 | 0.00 | 0.00 | 0.00 | 0.00 | 0.00 | 0.00 | 0.00 | 0.00 | 0.00 | 0.00 | 0.00 | 0.08 | 0.00 | 0.00 |  |  |
| A1 | 0.00 | 0.00 | 0.00 | 0.00 | 0.00 | 0.00 | 0.00 | 0.00 | 0.11 | -0.03 | 0.00 | 0.30 | 0.13 | 0.05 | 0.21 | 0.00 |  |
| A2 | 0.00 | 0.00 | 0.00 | 0.00 | 0.00 | 0.00 | 0.00 | 0.00 | 0.00 | 0.00 | 0.00 | 0.22 | 0.00 | 0.00 | 0.05 | 0.34 | 0.00 |
